# Human DNA ligase III bridges two DNA ends to promote specific intermolecular DNA end joining

**DOI:** 10.1093/nar/gkv652

**Published:** 2015-06-29

**Authors:** Vandna Kukshal, In-Kwon Kim, Gregory L. Hura, Alan E. Tomkinson, John A. Tainer, Tom Ellenberger

**Affiliations:** 1 Department of Biochemistry and Molecular Biophysics, Washington University School of Medicine, Saint Louis, MO 63110, USA; 2 Life Science Division, Lawrence Berkeley National Laboratory, Berkeley, CA 94720, USA; 3 Department of Internal Medicine and University of New Mexico Cancer Center, University of New Mexico, Albuquerque, NM 87131, USA; 4 Department of Molecular and Cellular Oncology, The University of Texas MD Anderson Cancer Center, Houston, TX 77030, USA

## Abstract

Mammalian DNA ligase III (LigIII) functions in both nuclear and mitochondrial DNA metabolism. In the nucleus, LigIII has functional redundancy with DNA ligase I whereas LigIII is the only mitochondrial DNA ligase and is essential for the survival of cells dependent upon oxidative respiration. The unique LigIII zinc finger (ZnF) domain is not required for catalytic activity but senses DNA strand breaks and stimulates intermolecular ligation of two DNAs by an unknown mechanism. Consistent with this activity, LigIII acts in an alternative pathway of DNA double strand break repair that buttresses canonical non-homologous end joining (NHEJ) and is manifest in NHEJ-defective cancer cells, but how LigIII acts in joining intermolecular DNA ends versus nick ligation is unclear. To investigate how LigIII efficiently joins two DNAs, we developed a real-time, fluorescence-based assay of DNA bridging suitable for high-throughput screening. On a nicked duplex DNA substrate, the results reveal binding competition between the ZnF and the oligonucleotide/oligosaccharide-binding domain, one of three domains constituting the LigIII catalytic core. In contrast, these domains collaborate and are essential for formation of a DNA-bridging intermediate by adenylated LigIII that positions a pair of blunt-ended duplex DNAs for efficient and specific intermolecular ligation.

## INTRODUCTION

DNA end joining by DNA ligase enzymes is essential for the replication, rearrangement and repair of DNA (1). Mammalian DNA ligases I, III and IV share a conserved, three-domain catalytic core that consists of the DNA binding domain (DBD), nucleotidyltransferase (NTase) domain and oligonucleotide/oligosaccharide binding domain (OBD) (2). Unique regions flanking the conserved core of each enzyme interact with proteins or DNA to support their specialized functions in DNA metabolism (2). While almost all eukaryotes have homologs of LigI and LigIV, LigIII is present in vertebrates but absent from ∼70% of eukaryotes, including *Saccharomyces cerevisiae* and *Schizosaccharomyces pombe*. Mammalian LigIII functions in both nuclear and mitochondrial DNA metabolism (3). Expression of the mitochondrial isoform of LigIII is essential for mammalian development and for cell growth under conditions requiring mitochondrial respiration (4–6). Mitochondrial DNA repair appears to be limited to a subset of the repair activities found in the nucleus (7). Mitochondrial LigIII is most strongly implicated in base excision repair (BER) and replication of mtDNA (8), but is not essential for cell viability in appropriate media (6). Nuclear LigIII forms a constitutive heterodimer with the repair scaffolding protein XRCC1, whereas mitochondrial LigIII functions independently of XRCC1 (9). A knockdown of LigIII expression sensitizes cells to oxidative damage and slows recovery from mtDNA depletion following DNA damage (10). Under normal growth conditions, knockdowns of LigIII have variable effects on steady-state levels of mtDNA that may depend on the extent of the knockdown (3,6). However, selective depletion of the mitochondrial isoform of LigIII alters the character of mtDNA replication intermediates, providing compelling evidence for the participation of LigIII in mtDNA replication (10). Finally, overexpression of mitochondrial LigIII protects cells against oxidative stress by increasing BER of damaged mtDNA, suggesting that ligation is the rate limiting step in mitochondrial BER (11).

In the nucleus, the LigIII/XRCC1 complex is recruited to damaged chromatin by the enzymatic activity of poly(ADP-ribose) polymerase 1 (PARP-1) (12). XRCC1 accelerates the repair of DNA single-stranded breaks in concert with LigIII (13) or DNA ligase I (14). However, LigIII can also function as a sensor of DNA strand breaks independently of PARP-1, serving to recruit other DNA repair proteins to sites of damage (15). LigIII has been implicated in the repair of DNA double strand breaks (DSBs) by microhomology-mediated end joining (16) or by a noncanonical pathway of DSB repair termed the alternative non-homologous end joining (A-NHEJ) pathway (17–19) when the classical Ku-dependent pathway (C-NHEJ) is inactivated by mutation. Cancerous cells overexpressing LigIII appear to be hyper-reliant on A-NHEJ for DSB repair (20–22), a phenotype that could be exploited for cancer therapy.

The unique N-terminal zinc finger (ZnF) domain of LigIII binds to DNA nicks or structural irregularities in duplex DNA (15,23–25). The ZnF also binds to PARP-1 (26) and this protein–protein interaction may facilitate DSB repair by the A-NHEJ pathway (27). The LigIII ZnF is homologous to the PARP-1 domains ZnF1 and ZnF2, which bind to DNA breaks and activate PARP-1 enzymatic activity (28). The isolated ZnF of LigIII binds weakly to nicked DNA and cooperates with the adjacent DBD to form a functional DNA binding module, the ZnF–DBD, with higher affinity than either the ZnF or DBD alone (29). In the context of the LigIII protein, the ZnF domain does not pack against the core catalytic domains (DBD–NTase–OBD) when the enzyme is bound to a nicked DNA (30). This was evident from the large radius of gyration (Rg) for LigIII constructs containing the ZnF, on or off DNA. The ZnF imbues the protein with characteristics of flexible or unfolded residues that are consistent with a highly extended or fluctuating conformation of the ZnF, disengaged from the DNA while the core of the enzyme encircles the nicked DNA (30). These findings have suggested that the ZnF binds only transiently to DNA single strand breaks, prior to end joining by the enzyme's catalytic core.

**Figure 1.**
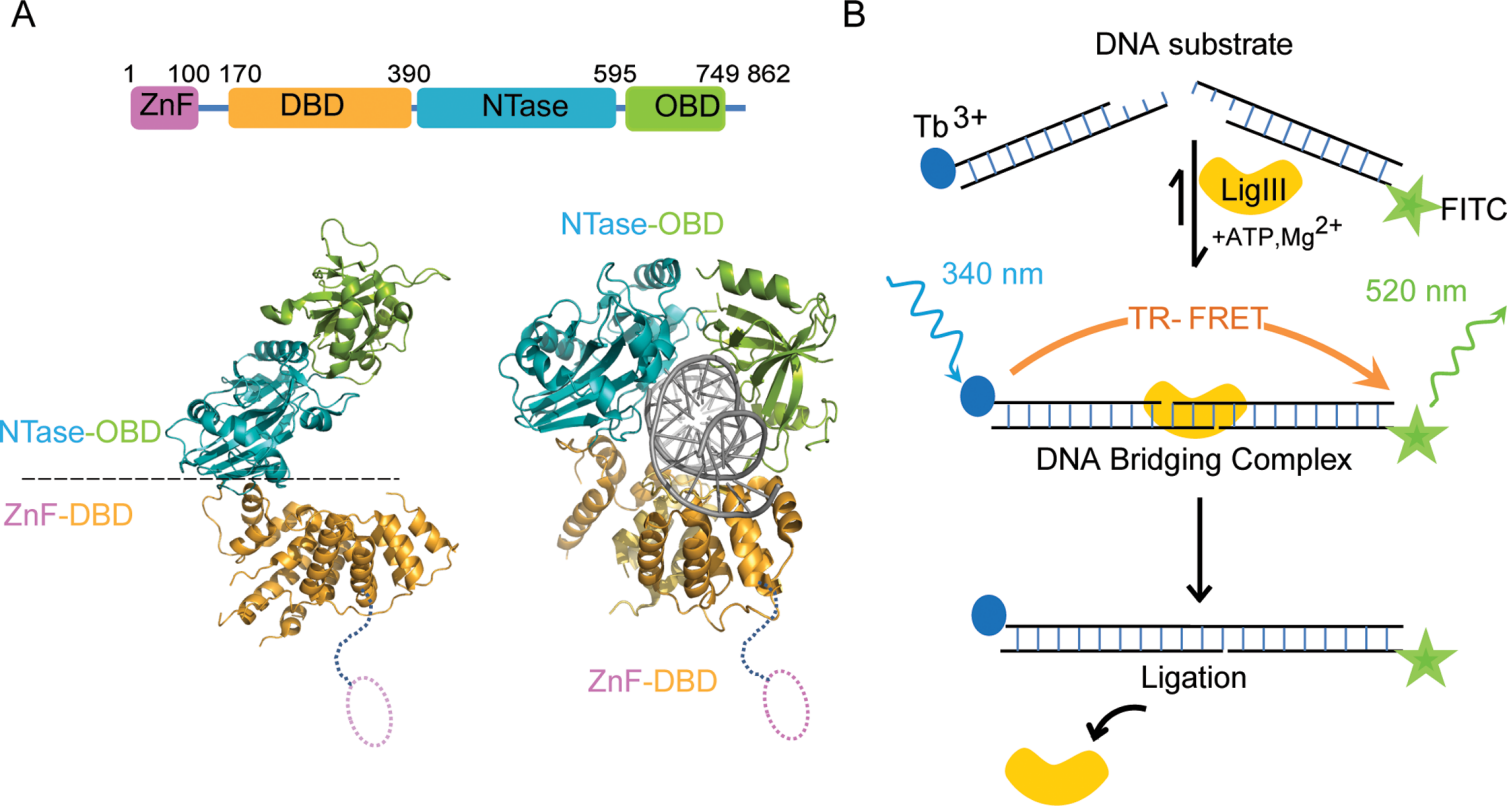
Tandem DNA binding surfaces of LigIII support DNA bridging. (**A**) The LigIIIβ protein (LigIII, 862 residues) spans four structural domains, an N-terminal zinc finger (ZnF; residues 1–100), a DNA binding domain (DBD; residues 170–390), a nucleotidyl transferase domain (NTase; residues 391–595) and the OB-fold domain (OBD; residues 596–749) that are required for efficient DNA end joining activity. Two pairs of domains, the ZnF–DBD and the NTase–OBD, comprise independent DNA binding modules (29) that are required in DNA bridging activity (*cf*. Figure 4B). The crystal structure of ΔZnF LigIII bound to a nicked DNA reveals a compact structure that would preclude the ZnF domain from binding to the nick (PDB 3L2P) (30). (**B**) We developed a time resolved FRET (TR-FRET) assay to measure intermolecular ligation of two DNAs by LigIII. In this assay, LigIII brings together DNAs labeled with Tb^3+^ (donor) and FITC (acceptor) to produce a FRET signal. DNA bridging activity and efficient intermolecular ligation of DNAs depend on the presence of the ZnF domain. We present evidence that this DNA bridging complex is a reaction intermediate of the intermolecular ligation pathway.

The ZnF–DBD is adjacent to another independent DNA binding module in LigIII consisting of the NTase and OBD domains. The NTase–OBD binds to duplex DNA and maintains a weak DNA nick joining activity in the absence of the Znf–DBD (29). Tryptophan quenching studies have revealed two DNA binding activities for LigIII with low and high affinity for duplex DNA (31) that may reflect the binding activities of the ZnF–DBD and NTase–OBD regions, respectively. Two adjacent DNA binding modules in LigIII could serve to align two DNA molecules for intermolecular ligation. Yet, a crystal structure of LigIII lacking the ZnF revealed a compact arrangement of the DBD–Ntase–OBD domains encircling a nicked DNA that excludes the ZnF from accessing the DNA nick, which is buried by the NTase and OBD domains (30). These results raise questions of when and how the ZnF engages DNA during LigIII-dependent repair of DNA strand breaks (see Figure 1A).

The ZnF has a modest effect on the DNA nick-joining activity of LigIII when assayed at high salt concentrations (32), but it is essential for efficient intermolecular ligation of two DNAs (30). To investigate the physical organization of two DNAs in complex with LigIII and the role of its ZnF, we developed a series of assays that reveal the presence of a stable and specific DNA bridging complex in which one molecule of LigIII closely apposes the ends of two DNAs prior to ligation. With this assay, we characterized a sustained binding activity of the ZnF, which assembles the enzyme–substrate complex for the intermolecular joining of two DNAs. We refer to this ligation reaction intermediate as the LigIII DNA bridging complex. This bridging complex requires LigIII adenylation, Mg^2+^ ions and matched DNA ends with no mismatches: consistent with the relevance of bridging to the DNA end joining reaction. Whereas the ZnF and OBD domains collaborate to bind two molecules of DNA in this bridging complex, there is a binding competition between these domains on a nicked DNA duplex. Taken together these results inform the distinct roles of LigIII domains in nick joining and NHEJ activities.

## MATERIALS AND METHODS

### DNAs and proteins

Oligonucleotides were synthesized on a 1 μmol scale using an Applied Biosystem 394 DNA-RNA synthesizer, HPLC purified and desalted using SepPak C18 cartridge (Waters). The 5′-modified oligonucleotides were prepared by incorporation of biotin, fluorescein isothiocyanate (FITC) and PO_4_ phosphoramidite monomers (Glen Research). To generate dsDNA substrates, equal amounts of complementary oligonucleotides were mixed in 25 mM MES (pH6.5) and 50 mM of NaCl. The oligonucleotide mixture was heated to 95°C and then allowed to anneal by slowly cooling to room temperature in a water bath. A 5′-biotinylated oligonucleotide (biotin-5′-AGTGAATTCGAGCTCG-3′) was annealed with a complementary oligonucleotide (5′-AATCGAGCTCGCAATTCACT-3′) to create a biotin labeled duplex with a 3-nt, 5′ overhang (underlined). The double-stranded DNA was labeled with the FRET donor terbium (Tb^3+^) by incubating the DNA with 10 nM Tb^3+^-streptavidin (Life Technologies; Figure 1) in the reaction mixture incubated at room temperature. A 5′ FITC labeled oligo (FITC-5′-CATGATTACGG-3′) was annealed with a complementary oligo (5′-ATTCCGTAATCATG-3′) to create a duplex with a 5′ overhang (underlined) that is complementary to the Tb^3+^ labeled DNA above. The blunt ended DNAs for time-resolved fluorescence resonance energy transfer (TR-FRET) and ligation assays were prepared with the same sequences listed above, but without the 3-nt overhanging ends. DNA substrates for enzymatic ligation were prepared by synthetic incorporation of a 5′-PO_4_ group in the unlabeled DNA strand of each duplex.

Full length DNA LigIIIβ (residues 1–862), which we refer to as LigIII throughout this manuscript, was cloned with an N-terminal His-tag in the pQE32 expression vector (Qiagen) and purified by a previously reported protocol (29). The ZnF deleted construct (ΔZnF LigIII; residues 170–749) was cloned with an N-terminal His-tag in pET28a (Novagen) and the construct deleted for the OBD domain (ΔOBD LigIII; residues 1–595) was cloned with C terminal-his tag in the pET28a vector (Novagen). ΔZnF LigIII was purified using a published protocol for the slightly larger ΔZnF LigIII spanning residues 170–862 (23). ΔOBD LigIII was expressed in *Escherichia coli* Rosetta 2 (DE3) cells at 16°C temperature. Cells were lysed by sonication and the soluble fraction was purified by Ni-NTA affinity chromatography. After washing the column with buffer A (50 mM Tris–HCl 7.5, 200 mM NaCl, 0.5 mM TCEP, 10% Glycerol and protease inhibitors (PMSF, benzamidine, leupeptin and aprotinin) containing 50 mM imidazole, the protein was eluted with buffer A containing 300 mM imidazole. The eluted protein was loaded on a heparin affinity column (GE Health care) in buffer A containing 100 mM NaCl then eluted with a gradient of 0.1 M to 1 M NaCl. Fractions containing a single band corresponding to the purified ΔOBD LigIII were pooled and loaded on Superdex 200 column (10/300 GL; GE Health Care) then eluted in buffer containing 50 mM Tris–HCl 7.5, 200 mM NaCl, 10% glycerol, 0.1 mM ethylenediaminetetraacetic acid (EDTA), 1 mM dithiothreitol (DTT). Purified proteins were concentrated to 30–40 mg/ml concentration and stored at −80°C.

### TR-FRET assay

**Figure 2.**
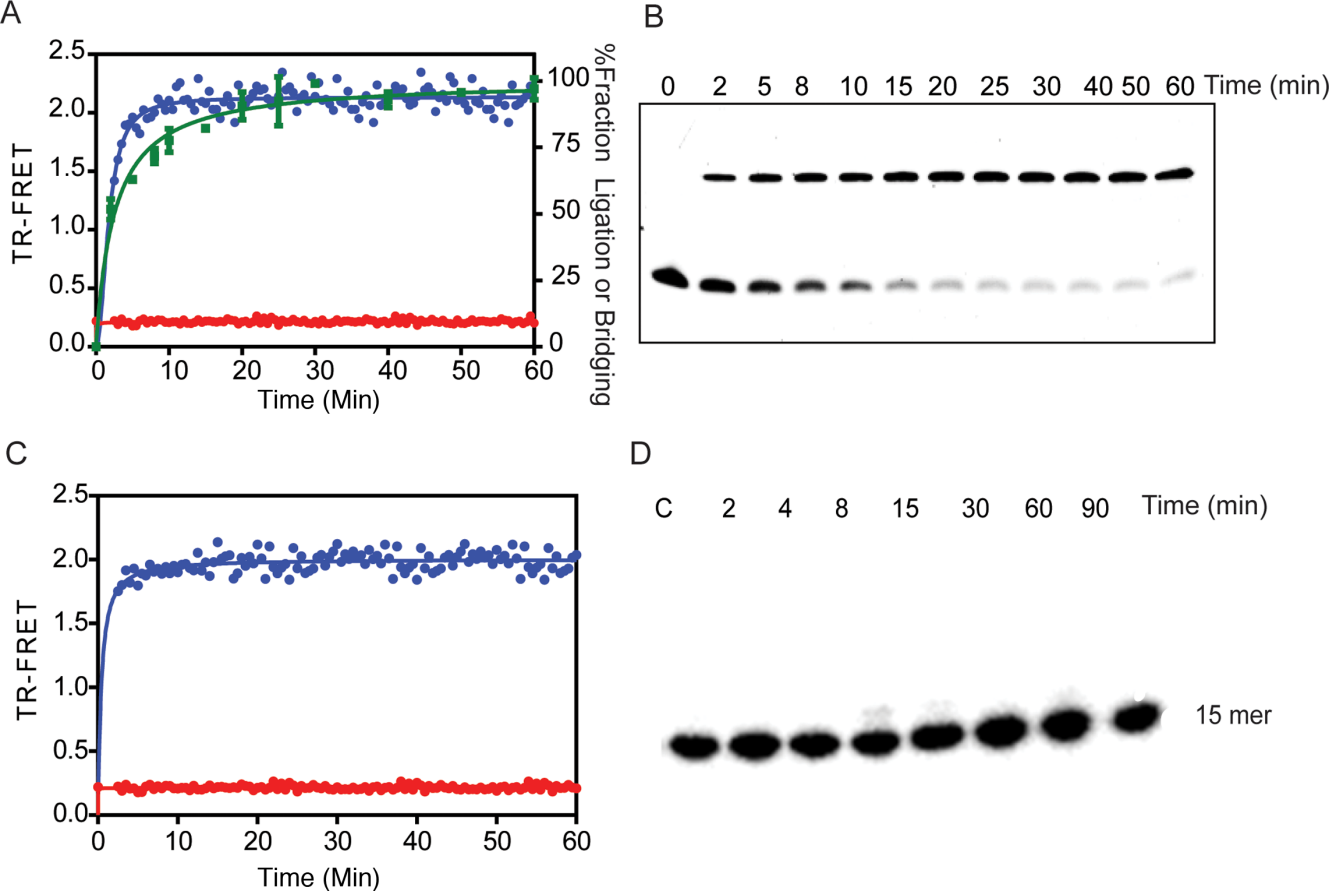
DNA bridging precedes intermolecular DNA ligation. (**A**) The real time measurement of TR-FRET (520/495 nm) signals the assembly of a DNA bridging complex, in which LigIII juxtaposes two DNAs labeled with donor and acceptor labels. Ligation reactions containing a pair of DNAs with complementary 3-nt overhangs were assembled in the absence (red) or presence (blue) of LigIII and the TR-FRET signal was monitored. The TR-FRET traces shown in Figures 2–5 are the average values of assays performed in triplicate. DNA ligation activity measured under the same reaction conditions (B) is plotted as the percentage of total DNA in the ligation product (green). (**B**) The time course of DNA ligation under the same reaction conditions as in (A) was monitored by running timed reaction aliquots on a 18% denaturing urea Polyacrylamide gelelectrophoresis (PAGE). DNA ligation activity is significantly slower than the TR-FRET signal, which results from the LigIII dependent juxtaposition of the labeled DNAs. The intermolecular ligation of a blunt-end DNA (Supplementary Figure S5) is markedly slower than ligation of overhanging DNA ends, suggesting that the end structure of the DNAs influences the rate-limiting step of ligation. (**C**) The same rapid onset of the TR-FRET signal is observed upon addition of LigIII (blue) to a pair of DNAs with 5′-OH groups that cannot be ligated, whereas the TR-FRET value remains at baseline in the absence of LigIII (red). Thus, DNA bridging complex formation is rapid and precedes DNA ligation (A). (**D**) PAGE analysis of the reaction with non-ligatable DNAs used in (C) shows that no ligated product is formed under conditions that produce the TR-FRET signal corresponding to DNA bridging activity.

**Figure 3.**
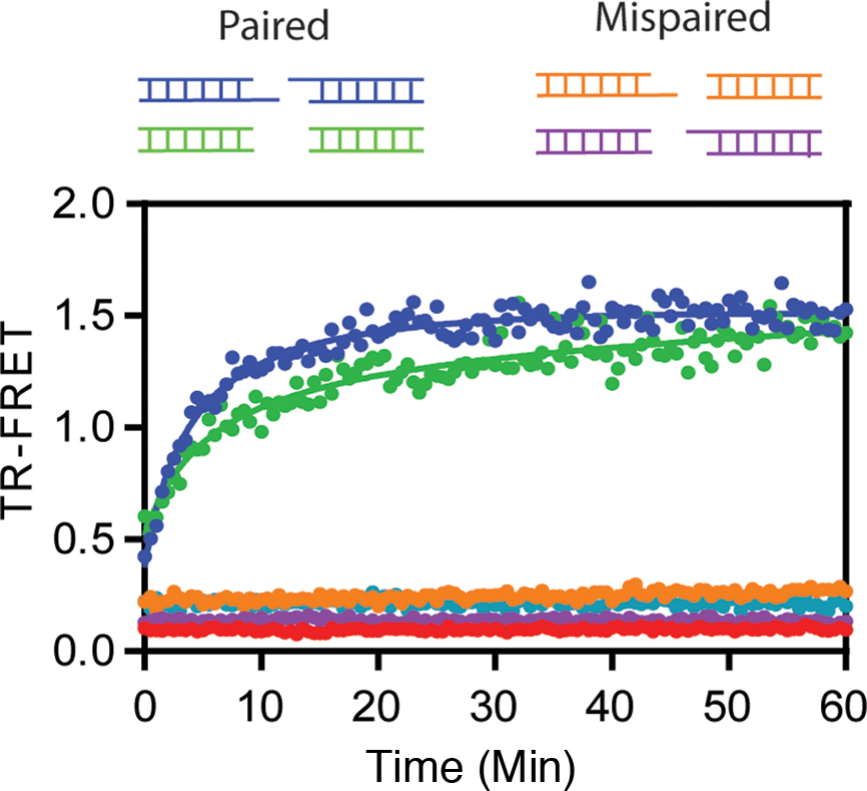
Bridging requires compatible pairs of DNA ends. Pairs of non-ligatable DNAs with either complementary three nucleotide overhangs (blue) or with blunt ends (green) generated a FRET signal in the presence of LigIII, indicative of DNA bridging activity. In contrast, two mispaired combinations consisting of two DNAs with blunt and overhanging ends (orange, magenta) did not support TR-FRET and resulted in a baseline level of fluorescence.

**Figure 4.**
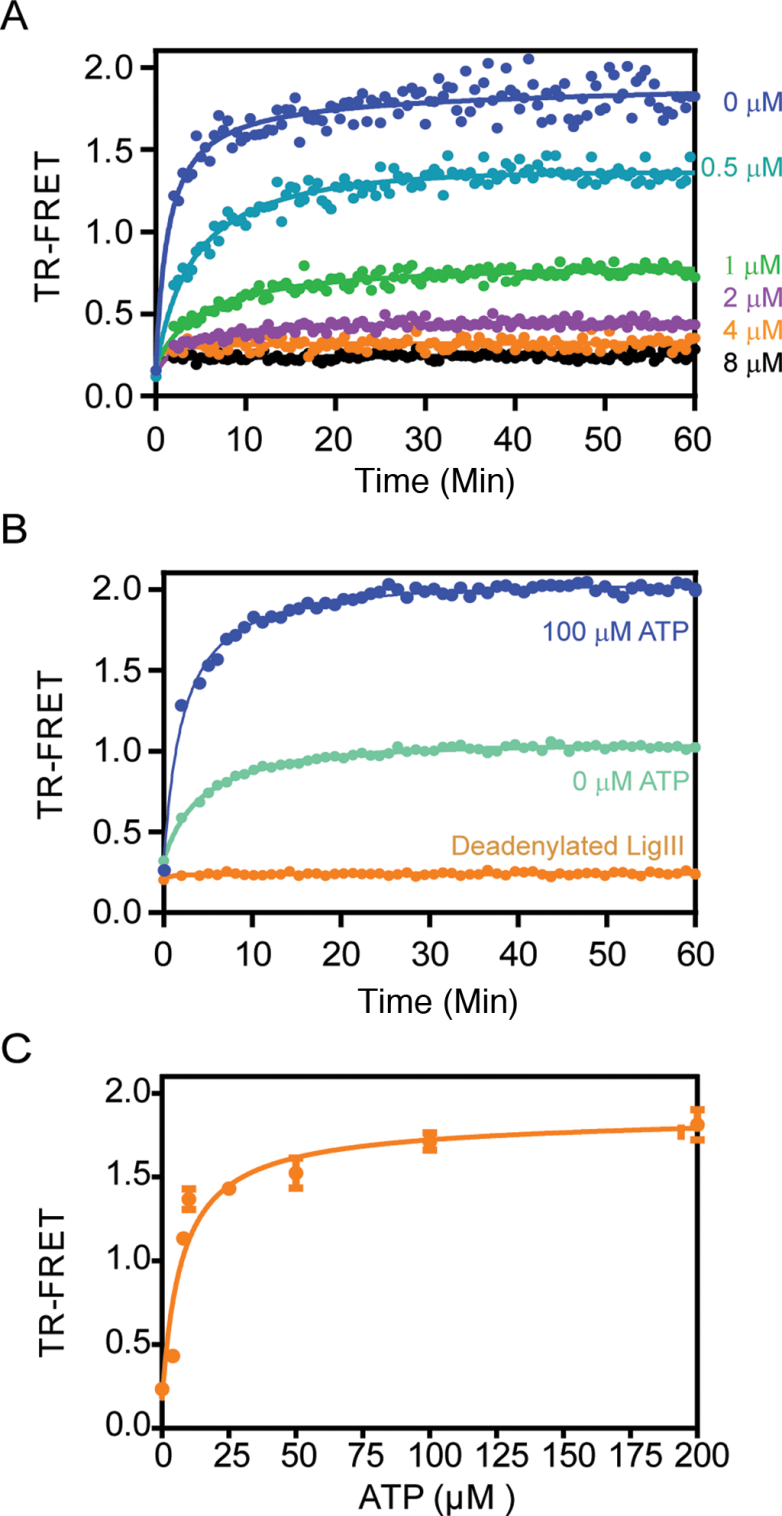
Specificity of DNA bridging by LigIII. (**A**) The DNA binding activity of LigIII in the DNA bridging assay is effectively competed by an unlabeled DNA, saturable manner, as expected for the formation of a discrete protein–DNA complex. A pair of labeled, non-ligatable DNAs (500 nM of each) was incubated with LigIII (100 nM) in the presence of increasing concentrations of an unlabeled DNA duplex (0–8 μM). See also Supplementary Figure S2. (**B**) In the absence of added ATP (light blue) the TR-FRET signal amplitude is about 50% of the signal for a reaction containing ATP (100 μM; dark blue). LigIII that was completely deadenylated (orange) did not support DNA bridging and TR-FRET. (**C**) Addition of ATP to the deadenylated LigIII (shown in B) restored the TR-FRET signal and DNA bridging activity in a concentration-dependent manner. The TR-FRET values 30 min after addition of ATP are shown.

**Figure 5.**
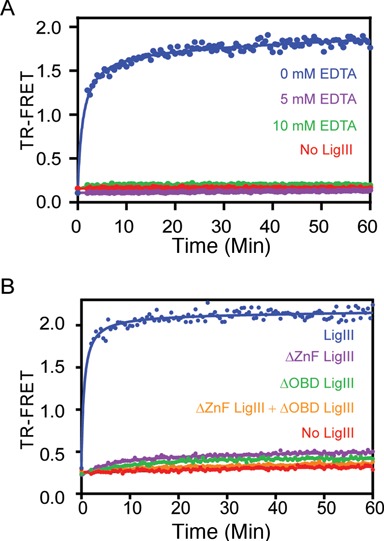
DNA bridging requires two independent DNA binding surfaces of LigIII. (**A**) Addition of EDTA (5 mM) abolishes the DNA bridging activity of LigIII in reaction buffer containing 5 mM MgCl_2_, indicating that divalent metal(s) are required for DNA binding activity. (**B**) Deletion mutants of LigIII lacking the ZnF (ΔZnF LigIII) or OBD (ΔOBD LigIII) domains do not form the DNA bridging complex although both mutants can bind to double-stranded DNA (Supplementary Table S1). A mixture of the ΔZnF and ΔOBD LigIII proteins also fails to bridge the labeled DNAs, consistent with a single molecule of LigIII mediating the DNA bridging activity (*cf*. Figure 7).

The time resolved FRET (TR-FRET) assay described by Chen *et al.* (33) was adapted to examine the DNA binding and bridging activity of LigIII. The TR-FRET assay was carried out in solid black 384-well flat bottom plates (Corning) and binding activity was monitored using a Synergy2 multi-plate reader (Bio-Tek). The binding reaction was initiated by mixing LigIII (100 nM) with a mixture of the Tb^3+^ and FITC labeled DNA duplexes (500 nM each) in a 20-μl reaction volume of TR-FRET buffer (50 mM HEPES-NaOH 7.5, 80 mM NaCl, 5 mM MgCl_2_, 1 mM DTT, 1 mM adenosine triphosphate (ATP) and 50 μg/ml bovine serum albumin; BSA) at room temperature. We confirmed that the order of component addition was inconsequential for the resulting TR-FRET signal. LigIII and the DNAs were stable in the assay conditions and could be preincubated separately for more than 1 h before mixing to initiate the binding reaction. For TR-FRET measurements, the Tb^3+^ donor was excited at 340 nm and, after a delay of 100 μs, the emission intensities of the Tb^3+^ donor (495 nm) and the FITC acceptor (520 nm) were recorded. The TR-FRET signal is expressed as an intensity ratio (520/495 nm). The time courses shown in Figures 2–5 are the average of three replicate binding reactions. A time delay between initiating the binding reaction and recording the TR-FRET data was accounted for in the data analysis. The amplitude of the TR-FRET signal is highly reproducible for replicate samples within an experiment, but varies (range of 1.5–2.5) according to the DNA labeling efficiency and the age of labeled DNAs. A limiting concentration of LigIII (100 nM) is used in our standard assay conditions in order to promote the formation of a 1:2 complex with one LigIII bound to two DNAs (*cf*. Figure 7B).

**Figure 6.**
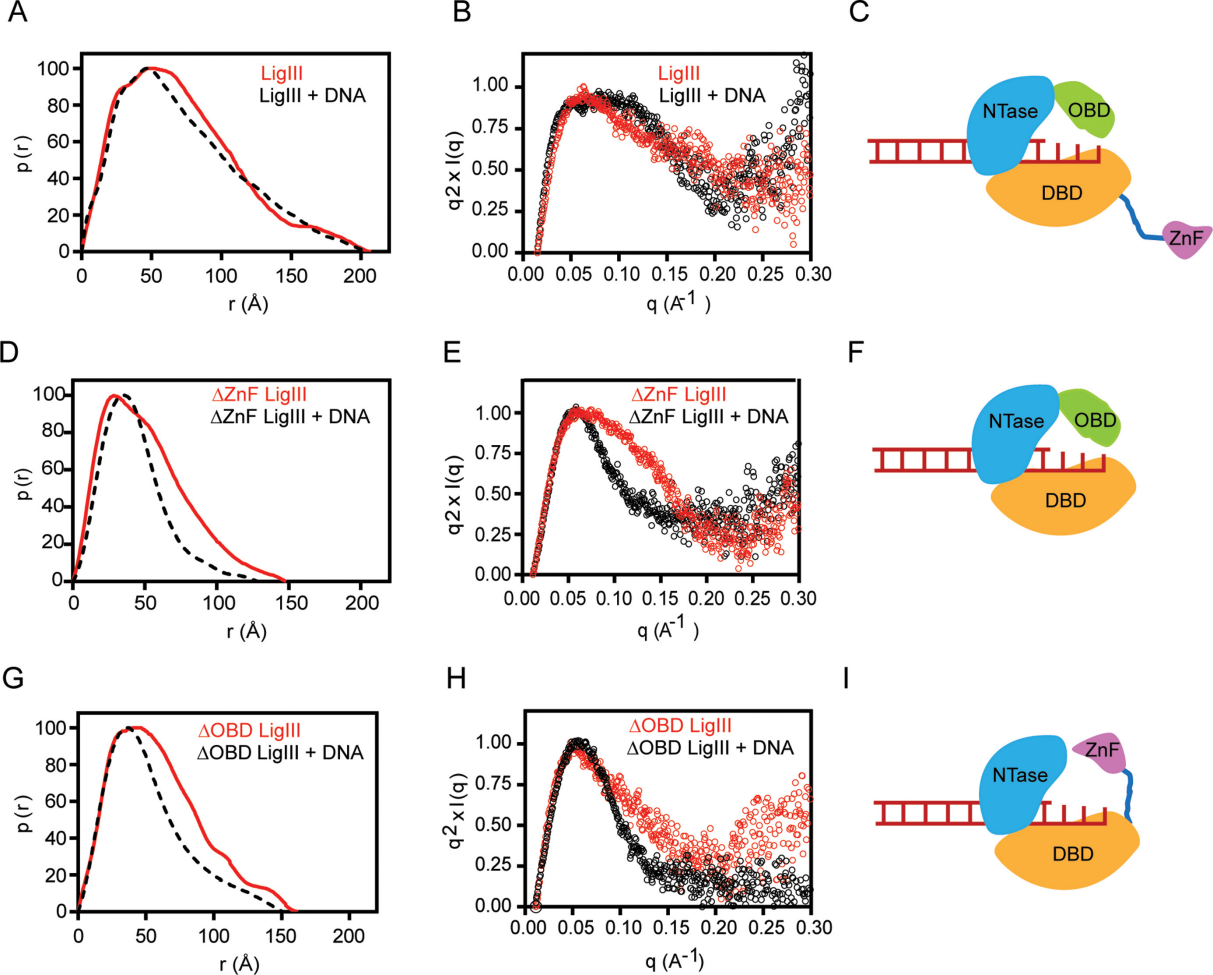
The ZnF and OBD domains bind to overlapping sites on DNA. Small angle X-ray scattering reveals the dynamic interactions of LigIII and deletion mutants in the 1:1 complex described in Figure 7A. (**A**) The normalized pair distribution function p(r) is consistent with an elongated conformation of LigIII in the presence or absence of a DNA duplex containing a three nucleotide overhang. (**B**) A normalized Kratky plot of the scattering data shows evidence of flexible residues in the presence or absence of DNA, which is modeled as a flexible ZnF domain in panel (**C**). (**D**) Deletion of the ZnF (ΔZnF LigIII) significantly narrows the p(r) distribution and reveals a large conformational transition upon binding to DNA. (**E**) The normalized Kratky plot of ΔZnF LigIII in complex with DNA shows a bell shaped Gaussian curve, indicative of a compact structure when bound to DNA. (**F**) The SAXS data are consistent with a model of ΔZnF LigIII encircling the DNA end as for the crystal structure of ΔZnF LigIII bound to nicked DNA (30). (**G**) Deletion of the C-terminal OBD domain (ΔOBD LigIII) narrows the pair distribution function p(r) of LigIII in a manner similar to deletion of the ZnF domain. This result suggests that the ZnF can engage DNA when the OBD is deleted, resulting in a compact conformation of ΔOBD LigIII bound to DNA. (**H**) A normalized Kratky plot of ΔOBD LigIII bound to DNA reveals a Gaussian curve consistent with a compact structure with the ZnF engaging the bound DNA as modeled in panel (**I**).

**Figure 7.**
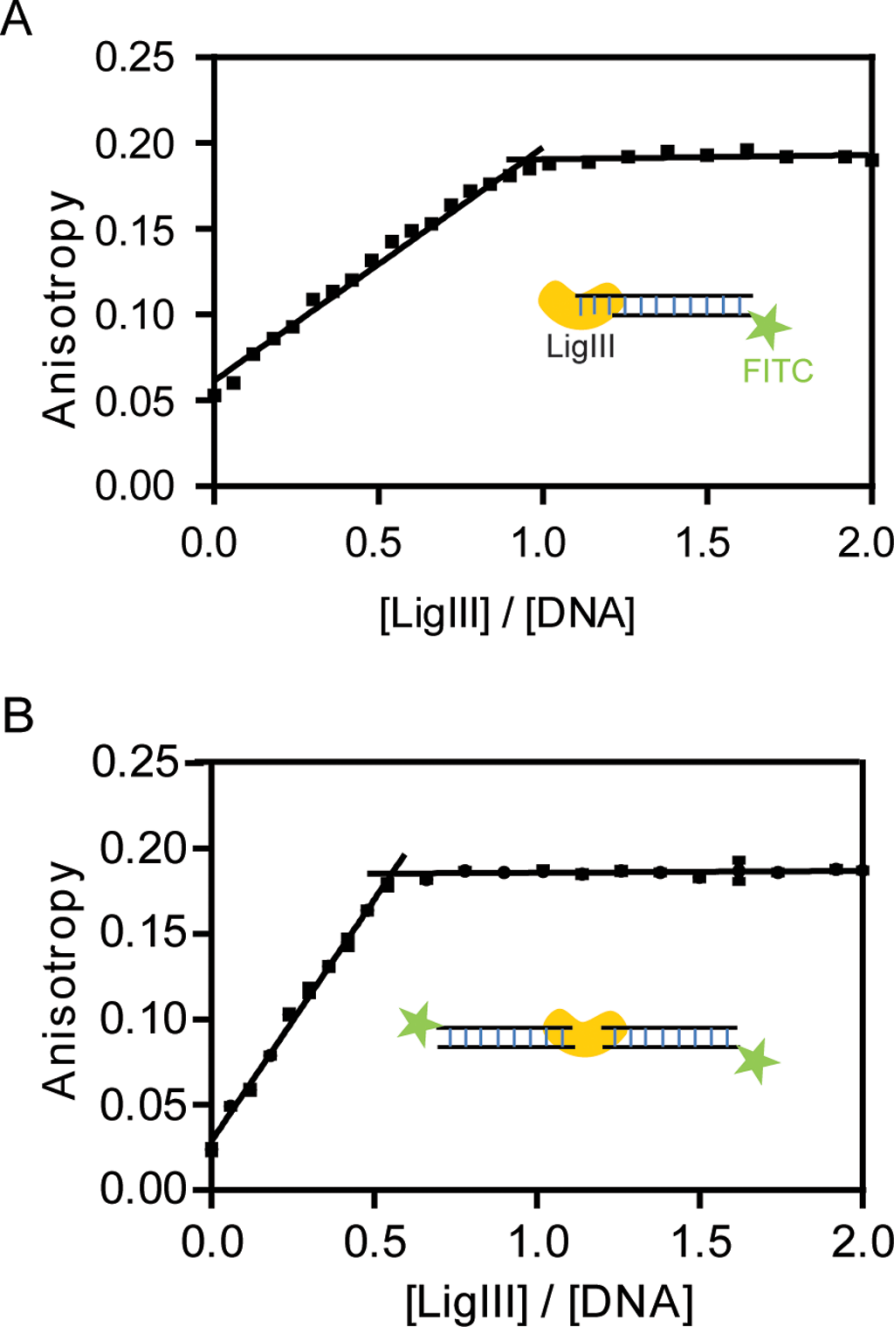
One molecule of LigIII binds two molecules of DNA in the bridging complex. (**A**) The DNA binding activity of LigIII was monitored by fluorescence anisotropy of a labeled DNA under conditions of stoichiometric binding. A 5′ FITC labeled duplex (800 nM) containing a three nucleotide overhang that cannot pair with itself was titrated with increasing concentrations of LigIII and the change in fluorescence anisotropy was measured. The DNA concentration in this assay is ∼5-fold higher than the apparent equilibrium binding constant (*K*_app_ ∼ 154 nM) determined with limiting concentrations of DNA and LigIII in excess. The measured binding stoichiometry of 1:1 for the LigIII:DNA complex was additionally confirmed by repeating the titration experiment at a higher concentration of DNA (1600 nM; not shown). (**B**) A binding stoichiometry of 1:2 (ratio = 0.5) was measured for LigIII in complex with a 5′ FITC labeled, blunt-ended DNA duplex (800 nM) that can form the DNA bridging complex. The same binding stoichiometry was determined in a binding reaction containing a higher concentration of the DNA (1600 nM; not shown).

### DNA end joining activity

Intermolecular DNA end joining activity catalyzed by LigIII was measured with DNA substrates containing complementary, 3-nt overhanging ends under multiple turnover conditions at 25°C. The reaction buffer and DNA sequences are the same as used in the TR-FRET assay, but the ligatable DNAs contain a 5′-PO_4_ on the unlabeled DNA strand, whereas the 5′ end of the other strand is blocked with FITC or biotin. The ligation reaction was initiated by mixing the DNA substrates (500 nM each) with LigIII (100 nM) in a 50 μl reaction volume. The time course of the ligation reaction was monitored by removing 10 μl aliquots and quenching activity with EDTA-formamide at 95°C for 5–10 min. The FITC labeled 11-mer substrate was separated from 30-mer ligation product by urea acrylamide gel electrophoresis under denaturing conditions. The substrate and product bands were visualized by fluorescence scanning with a Typhoon FLA 7000 image analyzer (GE Healthcare) using excitation at 495 nm and emission at 520 nm. The progress of the ligation reaction was measured from the fluorescence intensity of the product band and expressed as a percentage of the total fluorescence of the substrate and product bands in each sample.

### Pull-down of LigIII–DNA complexes

The DNA bridging complex was pulled down with a biotinylated DNA immobilized on streptavidin magnetic beads (Spherotech). Streptavidin magnetic beads (30 μl) were washed three times with 0.5 ml of wash buffer (10 mM HEPES-NaOH (7.0), 1 M NaCl, 5 mM DTT) then incubated with a nonligatable, 5′-biotinylated DNA duplex (500 nM) in a 100 μl reaction volume for 2 h at 4°C. Beads were then washed three more times with wash buffer by vortexing the beads, magnetic trapping of the beads and removal of the supernatant. The beads were subsequently blocked with wash buffer containing 50 μg/ml BSA for 30 min at 4°C then washed extensively with TR-FRET buffer prior to use. To form the DNA bridging complex, LigIII or ΔZnf LigIII (200 nM final concentration) was added to the washed beads in a 100 μl reaction volume and incubated for 30 min at 4°C. Beads were washed 4–5× with 0.5 ml TR-FRET buffer and a 5′-FITC labeled DNA duplex with non-ligatable ends (500 nM) was added in a 100 μl reaction volume and incubated for 30 min at 4°C. Beads were subsequently washed 3–4× with TR-FRET buffer and the 5′-FITC DNA was eluted from the beads by addition of 1% sodium dodecyl sulphate solution (40 μl). The amount of FITC-labeled DNA recovered from the beads was quantified by fluorescence intensity measured at 520 nm in solution or by running samples on 18% Urea polyacrylamide gelelectrophoresis and imaging the 5′-FITC oligos with the Typhoon imager, as described above for the DNA ligation assay. The specificity of binding was tested in control reactions lacking the immobilized, biotinylated DNA duplex.

### DNA binding affinity and stoichiometry determinations

The DNA binding affinities of different LigIII constructs were determined using non-ligatable, FITC labeled DNAs containing either a 3-nt overhang or a blunt end. The DNAs (25 nM) were titrated with LigIII proteins (0–1000 nM) in a 200 μl reaction volume of TR-FRET buffer. The decrease in DNA concentration caused by addition of different volumes of LigIII was taken into account in the analysis of the data. Samples were incubated in a 3 mm cuvette incubated at 25°C and fluorescence anisotropy was measured with a QuantaMaster C-60 spectrofluorimeter (Photon Technology International) using excitation and emission wavelengths of 495 and 520 nm, respectively. The apparent equilibrium dissociation constants (*K*_app_) were calculated by fitting the data to Equation (1) shown below (34):

(1) \begin{equation*} r = r^0 + (r_{\max } - r^0 )*f \end{equation*}

where r is the fluorescence anisotropy determined experimentally and

(2) \begin{equation*} f = p*\frac{d}{{[{\rm DNA}] + K_{{\rm app}} }} \end{equation*}

(3) \begin{equation*} p = [{\rm protein}]/\left( {1 + \frac{d}{{K_{{\rm app}} }}} \right) \end{equation*}

(4) \begin{equation*} \begin{array}{l} d = \\ \frac{{ - \frac{{[{\rm protein}]}}{{K_{{\rm app}} }} + \frac{{[{\rm DNA}]}}{{K_{{\rm app}} }} - 1 + \sqrt {\left( {\frac{{[{\rm protein}]}}{{K_{{\rm app}} }} - \frac{{[{\rm DNA}]}}{{K_{{\rm app}} }} + 1} \right)^2 + 4*\frac{{[{\rm DNA}]}}{{K_{{\rm app}} }}} }}{{\frac{2}{{K_{{\rm app}} }}}} \\ \end{array} \end{equation*}

To determine the stoichiometry of DNA binding by LigIII, the florescence anisotropy experiment was repeated at DNA concentrations more than four-fold above the experimentally determined *K*_app_ values (Supplementary Table S1). The DNAs (800 or 1600 nM) were titrated with LigIII (0–3 μM) in 200 μl of TR-FRET buffer and the change in fluorescence anisotropy was measured in three independent experiments for each titration. The mean values are shown in Figure 7, and in all cases, the standard deviations were <5% of the mean values.

**Figure 8.**
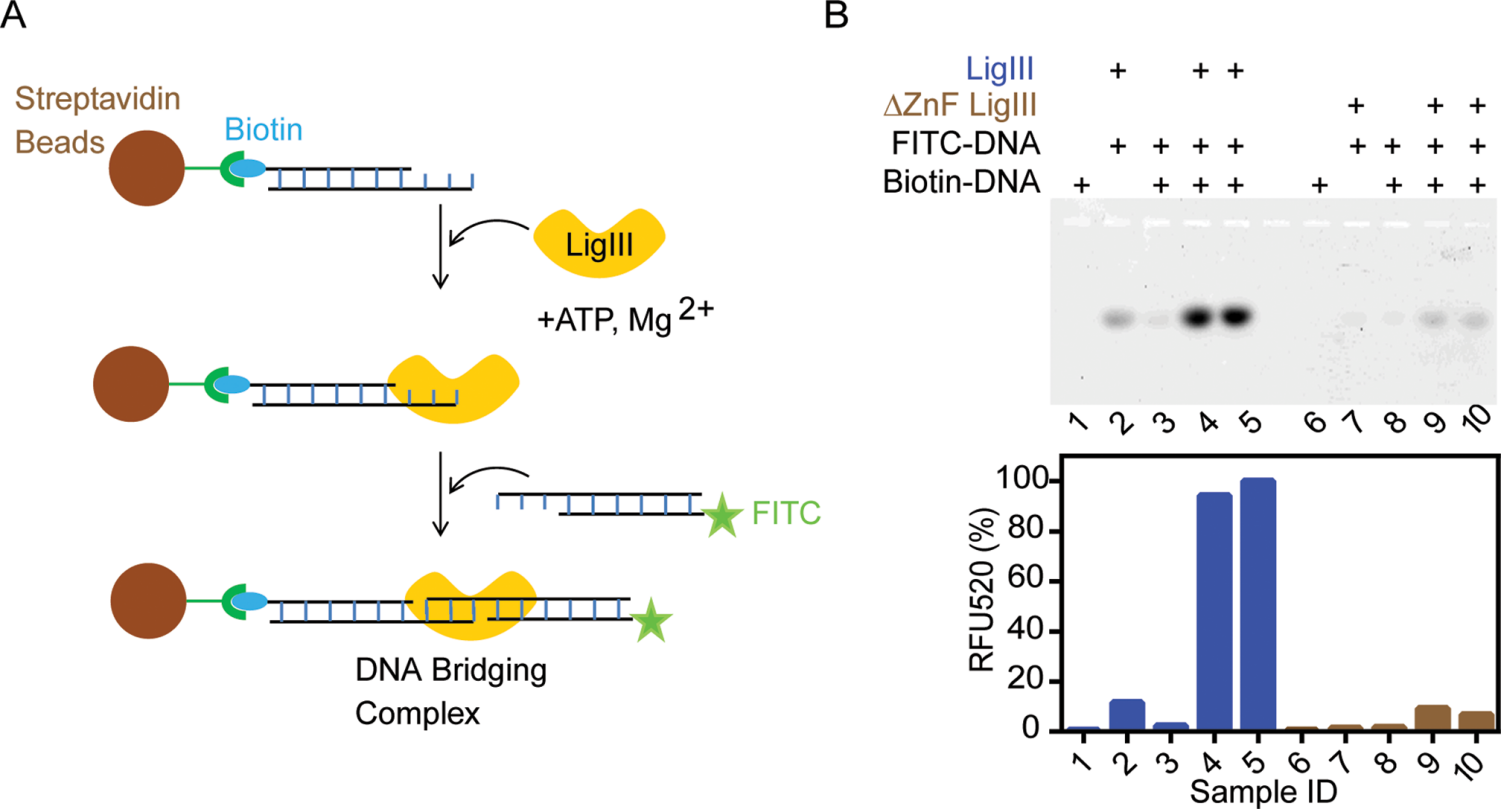
LigIII is stably bound to two DNAs in the bridging complex. (**A**) Streptavidin magnetic beads coated with a 5′ biotinylated DNA duplex were used to capture LigIII together with a 5′ FITC labeled DNA in a pull-down assay. The FITC labeled DNA on the beads was analyzed by urea PAGE and quantification of fluorescence emission intensity in the gel (see ‘Materials and Methods’ section). (**B**) Upper panel, denaturing urea PAGE analysis of the FITC labeled DNA recovered from the beads. Lower panel, quantitation of fluorescence intensity in the gel. Samples 4 and 5 are replicates containing LigIII in a bridging complex. Samples 9 and 10 are replicates containing the ΔZnF LigIII mutant protein, which does not form the bridging complex. Figure 8 shows the pull-down of DNAs with complementary 3 nt overhangs in complex with LigIII. Additional controls are shown in the legend above the gel. Supplementary Figure S3 shows the pull-down of a LigIII DNA bridging complex incorporating two blunt-ended DNAs.

### Small angle X-ray scattering

LigIII and ΔZnF LigIII were adenylated with 5 mM of MgCl_2_ and 1 mM of ATP for 2 h at 4°C then the reaction was stopped by adding 10 mM of EDTA. Proteins were dialyzed with a buffer containing 50 mM HEPES-NaOH (7.5), 80 mM NaCl, 1 mM EDTA, 5 mM DTT and 10% glycerol. For protein–DNA complexes different versions of LigIII were incubated with non-ligatable (5′-OH) nicked and 3 nt overhang at a 1:3 ratio of protein to DNA. LigIII was bound to a DNA with a non-self complementary overhang in the buffer described above. The protein–DNA complex was purified on a Superdex 200 10/300GL (GE healthcare) size exclusion column and the presence of DNA in the complex was confirmed by the absorbance ratio of A_260_: A_280_. Attempts to examine the DNA bridging complex using SAXS were unsuccessful because of sample aggregation at the micromolar concentrations of protein and DNA required for SAXS.

SAXS data for LigIII in complex with a non-bridging DNA were collected at beamline 12.3.1 of the Advanced Light Source, Lawrence Berkeley National Lab (Berkeley, CA, USA) (35,36). Incident X-rays were tuned to a wavelength (λ) of 1.03 Å at a sample-to-detector distance of 1.5 m resulting in scattering vectors (q) ranging from 0.007 to 0.31 Å^−1^. The scattering vector is defined as *q* = 4π sin(θ/λ), where 2θ is the scattering angle. All experiments were performed at 20°C and data were processed as described previously (35). The SAXS data for different protein concentrations were examined for aggregation using Guinier plots (37), in order to choose suitable samples for further analysis. The radius of gyration R_G_ was derived by the Guinier approximation I_(q)_ = I_(0)_ exp (−q^2^ R_g_^2^/3) with the limits q·R_g_ < 1.3. The program GNOM (38) was used to compute the pair-distance distribution functions, P(r) and D_max_. Kratky plots (q^2^ x I_(q)_ versus q) of each dataset were prepared to assess the globular or Gaussian chain-like nature of the protein in solution.

## RESULTS

### Formation of a DNA bridging complex

In contrast to mammalian DNA ligases I and IV, LigIII can efficiently join two DNAs with cohesive or blunt ends, without the use of molecular crowding agents like PEG (39,40). The unique N-terminal ZnF domain of LigIII contributes strongly to blunt end DNA ligation activity (23,29) by an unknown mechanism. LigIII contains four domains that can open or close around dsDNA (Figure 1A). Here, we characterized the assembly of the corresponding enzyme–substrate complex, using a TR-FRET assay (33). Our assay incorporates two double-stranded DNAs with cohesive or blunt ends (see ‘Materials and Methods’ section). One strand of each DNA duplex in the reaction is labeled on a 5′ end either with a biotin that is conjugated with Tb^3+^-labeled streptavidin (the FRET donor) or with the FRET acceptor FITC (Figure 1B). Upon addition of LigIII to a pair of DNAs with ligatable ends, the TR-FRET signal increases rapidly and reaches a maximum within minutes (Figure 2A). The increase in TR-FRET occurs faster than the rate of DNA ligation measured under the same conditions with the labeled DNAs (Figure 2A and B). This observation suggested that the TR-FRET signal is reporting on a LigIII dependent activity in which two DNA molecules are brought into apposition before intermolecular ligation. In fact, substituting a pair of non-ligatable DNA molecules with 5′-OH and 3′-OH ends produced the same rapid increase in FRET upon addition of LigIII (Figure 2 C) without formation of ligation product (Figure 2D). We therefore refer to this early step of the reaction, prior to covalent ligation of DNAs, as a DNA bridging complex. A limiting concentration of LigIII is used in the bridging assay to promote the formation of a 1:2 complex of one LigIII molecule bound to two DNAs (see below). The TR-FRET signal obtained in the bridging assay (TR-FRET = 1.5–2.0) is correspondingly less than the maximum value (TR-FRET = 3.0) measured for a control DNA that incorporates the FRET donor and the acceptor on the 5′ ends of a DNA duplex (Supplementary Figure S1A).

The DNA binding specificity of LigIII in the bridging complex was investigated using DNAs that lack a 5′-PO_4_ and cannot be ligated together. DNA ligase I and LigIII both discriminate strongly against ligation substrates with mispaired bases located near the 5′-PO_4_ and 3′-OH ends of a DNA strand break at physiological salt concentrations (32,41). This substrate selectivity for correctly paired bases at the ends enhances the fidelity of DNA ligation by preventing the aberrant joining of DNA breaks with unmatched overhangs (29,41). Here we found that the formation of the DNA bridging complex also requires matched DNA ends. Addition of LigIII to a pair of labeled DNAs with blunt ends or complementary 3 nt overhangs generates the FRET signal, whereas a mixture of DNAs with unmatched ends does not support the DNA bridging interaction (Figure 3). This finding suggests that the ends of the DNAs are in close contact with one another in the bridging complex, as expected for a productive enzyme–substrate complex. The specificity of the DNA bridging complex was further tested by a competitive binding reaction with unlabeled DNA. The TR-FRET signal is completely blocked by addition of an eight-fold excess of unlabeled DNA (4 μM) to a reaction containing 0.5 μM of each labeled DNA (Figure 4A). The observed binding competition is consistent with a discrete and saturable DNA binding surface of LigIII that maintains the bound configuration of DNAs in the bridging complex. The rate of dissociation of the labeled DNA from the bridging complex (0.1 min^−1^; Supplementary Figure S2 and Methods) was estimated in the presence of a saturating concentration of an unlabeled competitor.

### Adenylation of LigIII is essential for bridging complex formation

During the first chemical step of DNA ligation, LigIII is adenylated on an active site lysine (Lys 421 in human LigIII) using ATP as the substrate. Recombinant LigIII protein purified from *E. coli* consists of an approximately equal mixture of adenylated and unmodified protein molecules (30). Correspondingly, the TR-FRET signal amplitude generated in the absence of added ATP is about 50% of the signal amplitude in the presence of a saturating amount of ATP (Figure 4B), implying that unmodified LigIII does not support DNA bridging activity. We confirmed this conclusion using unmodified LigIII, which was prepared by incubation with inorganic pyrophosphate to reverse the adenylation chemistry then exchanged into our standard assay buffer by gel filtration chromatography. Deadenylated LigIII does not support TR-FRET activity (Figure 4B), but addition of ATP to the deadenylated enzyme fully restores bridging activity (Figure 4C). The adenylation status of LigIII does not affect DNA binding activity towards a single DNA duplex (Supplementary Figure S4), consistent with the independent DNA binding activity of the ZnF–DBD that is unaffected by adenylation of the NTase domain. The DNAs used in this reaction contain a 5′-OH end instead of a 5′-PO_4_, which prevents transfer of the adenylate group from LigIII to the 5′-PO_4_ of the DNA (step 2) and subsequent ligation of the bound DNAs (step 3). We conclude that adenylation of LigIII, and not the subsequent transfer of the adenylate to DNA, is required for DNA bridging activity. The prerequisite for adenylation of LigIII supports the relevance of bridging to the DNA end joining reaction. It is reminiscent of the loss of DNA binding activity caused by mutation of lysine-adenylate acceptor residue in viral DNA ligases (42,43).

Magnesium ions are essential for three chemical steps of the ligation reaction (1). At saturating concentrations of Mg^2+^, the efficiency of ligation is high and all three steps of the reaction occur at similar rates. However, under conditions of limiting Mg^2+^ the final step of DNA phosphodiester bond formation becomes rate-limiting and the 5′-adenylated DNA intermediate accumulates (44). The addition of 5 mM EDTA completely abolishes DNA bridging complex formation by LigIII (Figure 5A), showing the importance of Mg^2+^ ions for the stable interaction of adenylated LigIII with two complementary DNAs.

### The ZnF and OBD domains of LigIII are both required for DNA bridging

We next examined the roles of LigIII domains other than the adenylation domain in DNA bridging activity. The ZnF domain of LigIII is described as a DNA nick-sensor (23,32) that contributes only modestly to the ligation of DNA single strand breaks *in vitro* (29) The ZnF is not required to complement LigA-deficient *E. coli* (32) or to rescue the lethality of knocking out the mitochondrial isoform of LigIII in mammalian cells (4). However, the ZnF domain is required for the highly efficient, intermolecular DNA ligation activity that is a hallmark of LigIII (23,29). Accordingly, a deletion of the ZnF domain completely abolishes the formation of the bridging complex (Figure 5B), implicating the ZnF–DBD as one of the key DNA interaction surfaces in the complex.

The OBD is a conserved domain of mammalian DNA ligases with dual roles in promoting the step 1 adenylation reaction (17) and for DNA binding activity in cooperation with the adjacent NTase domain (30). Deletion of the OBD eliminates the DNA bridging activity of LigIII (Figure 5B), which implicates the NTase–OBD as the second DNA binding module in the bridging complex. The ΔZnF-LigIII and ΔOBD-LigIII retain DNA binding activity (Supplementary Table S1) but fail to support DNA bridging individually or in a 50:50 mixture of the mutant proteins (Figure 5B). The requirement for the ZnF and OBD domains *in cis* suggests that LigIII uses both of these DBDs in the bridging complex. Correspondingly, human DNA ligase I, which is homologous to LigIII but lacks a ZnF domain, does not exhibit DNA bridging activity (Supplementary Figure S1B).

### Structural dynamics of LigIII DNA binding modules

Mammalian DNA ligases are flexible, multidomain proteins that adopt an elongated shape in the absence of DNA (45,46) and a compact, ring-shaped structure in complex with a nicked DNA (30,34). However, small angle x-ray scattering (SAXS) data for LigIII are consistent with a highly elongated shape even when bound to DNA (Figures 1A and 6A) (30). Kratky plots of the x-ray scattering data highlight the flexibility of LigIII on and off of DNA, exhibiting non parabolic behavior with longer decays at high q-values (Figure 6B). Deletion of the ZnF domain sharpens the pair distribution function p(r) of the mutant LigIII in complex with DNA (Figure 6D) (30), and the Kratky plot for this complex exhibits a near parabolic shape indicative of a compact or globular protein (Figure 6E). These results suggest that the ZnF domain is flexible and not stably bound to a nicked DNA, whereas the DBD, NTase and OBD domains bind in a compact arrangement consistent with the crystal structure of this complex (30). In the crystal structure of ΔZnF LigIII, the DNA nick is buried by the NTase and OBD domains and this would appear to prevent the ZnF domain from binding to the DNA nick (30).

We reasoned that the ZnF and OBD domains might compete for binding to DNA ends, and perhaps bind sequentially to DNA during a ligation reaction. To examine possible alternative binding modes of the ZnF and OBD domains, we compared the SAXS profiles of ΔZnF LigIII and ΔOBD LigIII in the absence and presence of DNA containing a 3 nt, non-self complementary overhang (Figure 6) or a nicked DNA duplex (Supplementary Table S2). The ΔZnF and ΔOBD LigIII proteins show a comparable sharpening of their p(r) distributions in comparison to intact LigIII, when bound to DNA. Kratky plots confirm that the ΔZnF and ΔOBD LigIII mutants exhibit less residual flexibility than intact LigIII when bound to DNA (Figure 6). These results show that the DNA binding activity of the ZnF domain is modulated by the OBD, with overlapping binding sites on a nicked DNA or an unpaired DNA with a non-self-complementary overhang. This binding competition is eliminated by deletion of the ZnF or the OBD, and both of these deletion mutants assemble into a compact structure when bound to DNA (Figure 6D-I). However, the ZnF and OBD cooperate rather than compete when two DNAs interact with LigIII in a bridging configuration, requiring both of these domains as well as the DBD and NTase domain (Figure 5B).

### One molecule of LigIII bridges two DNA molecules

The DNA binding stoichiometry of LigIII was measured for a blunt-ended, nonligatable DNA under conditions that support bridging complex formation and for a DNA with an overhanging end that cannot self-pair and does not support DNA bridging activity. DNA binding activity was monitored by fluorescence anisotropy using high concentrations of the FITC-labeled DNAs (800 or 1600 nM) in order to promote stoichiometric binding by LigIII (*K*_DNA_ of ∼150 nM; Supplementary Table S1) (29). For the unpaired DNA with an overhang, the DNA binding activity of LigIII saturates at a 1 : 1 molar ratio of protein:DNA (Figure 7A). For the blunt-ended DNA that forms a bridging complex (Figure 7B), the binding activity of LigIII saturates at a 0.5:1 ratio corresponding to one molecule of LigIII bound to two DNAs in the bridging complex (Figure 7B).

### The DNA bridging complex is stable

The physical stability of the DNA bridging complex with LigIII was assessed by a pull-down assay. A 5′-biotinylated dsDNA was immobilized on streptavidin-coated magnetic beads and the beads were incubated with LigIII and subsequently with a FITC-labeled DNA (Figure 8A). The DNAs in this assay have compatible overhangs but lack a 5′-PO_4_ and cannot be ligated together. DNA bridging activity was measured by eluting the FITC-labeled DNA from the beads after extensive washing to remove unbound DNA. LigIII efficiently captured the FITC-labeled DNA on beads with an immobilized, complementary DNA, but not on beads lacking the immobilized DNA (Figure 8A). The ΔZnF LigIII did not support DNA bridging activity in this assay (Figure 8B) in agreement with the TR-FRET assay results (Figure 5B). This experiment was repeated with blunt-ended DNAs, which are also efficiently pulled down in the DNA bridging complex (Supplementary Figure S3). However, a pair of mismatched DNAs consisting of a FITC-labeled, blunt-ended DNA and an immobilized DNA with an overhanging end did not show bridging activity in this assay (data not shown). These pull-down experiments reveal a stable complex between LigIII and two DNAs with blunt ends or matching overhangs, as for an intramolecular ligation reaction.

## DISCUSSION

The existence of multiple mammalian DNA ligases makes it important to understand their distinct features that allow them to function in different replication and repair pathways (1). LigIII is therefore of special interest for its functions in multiple pathways and for its ZnF domain that flanks the conserved, three-domain ligase core (2). In particular, the efficient joining of duplex DNAs by LigIII portends a mechanism for aligning two DNA substrates in the enzyme active site. We previously showed that LigIII contains two independent DNA binding modules corresponding to the ZnF–DBD and the NTase–OBD (29). Here we show these dual DNA binding modules contribute to the assembly of a stable complex with one molecule of LigIII bound to two molecules of DNA. The resulting DNA bridging complex closely apposes two compatible DNA ends for ligation, either blunt ends or short overhangs that can base pair. The DNA bridging complex with LigIII is remarkably long-lived for an enzyme–substrate complex and can be pulled down with an immobilized DNA. Modification of the LigIII active site with an adenylate group, catalyzed during the first step of the three-step ligation reaction, is required for bridging in the equilibrium binding condition for TR-FRET and in the pull-down experiment. This again places the bridging complex in the reaction pathway for intermolecular ligation, as a precursor to DNA end joining.

The unique ZnF domain of LigIII is required for bridging between two DNA molecules in addition to its previously characterized DNA nick-sensing function (15,32,47). This finding underscores the importance of the ZnF for intermolecular DNA joining activity (29,47). The DNA binding specificity for assembly of the bridging complex assembly is suggestive of a function for the ZnF in holding two DNA substrates in close apposition prior to intermolecular ligation, raising the possibility that the ZnF-dependent DNA bridging activity of LigIII contributes to the repair of DSBs in mammalian cells by A-NHEJ in the nucleus (48) and possibly during the replication or repair of mtDNA (11,18). It is notable that the rate of intermolecular DNA ligation lags only slightly behind the rate of bridging complex assembly for DNAs with overhanging ends (Figure 2A). The rate of blunt end DNA ligation is much slower than assembly of the bridging complex (Supplementary Figure S5), which suggests that correct alignment of the DNA ends within the bridging complex may be rate-limiting for ligation activity.

We have developed a fluorescence-based assay monitoring the juxtaposition of two DNAs by LigIII in a manner consistent with an intermolecular DNA ligation reaction. This real-time assay performed in a multi-well format is suitable for high-throughput screening for inhibitors, which may be useful tools to understand the biological functions of the unique ZnF domain of LigIII and other aspects of the DNA joining reactions catalyzed by LigIII. Here the assay identifies the formation of a DNA bridging complex that aligns two DNAs for covalent ligation. Assembly of the bridging complex requires blunt-ended duplex DNAs or complementary overhangs that can base pair, plus adenylation of the active site Lys 421. It may therefore prove useful scientifically or medically to employ this or other assays to develop small molecules that target the bridging complex by blocking the ZnF conformation or other aspects of the complex assembly as well as the LigIII active site chemistry. Furthermore, the role of the ZnF in forming stable and specific DNA bridging complex in which one molecule of LigIII closely apposes the ends of two DNAs prior to ligation suggests that the ZnF and the bridging complex identified here provides a specificity step prior to the ligation chemistry. The ZnF thus likely contributes to both specificity and efficiency of DNA LigIII in joining separate DNAs with compatible ends.

## Supplementary Material

SUPPLEMENTARY DATA
