# Supplementary material for: Human DNA ligase III bridges two DNA ends to promote specific intermolecular DNA end joining
Source: Nucleic Acids Res. 2015 Jun 29;43(14):7021–31. doi: 10.1093/nar/gkv652 (PMC4538836; doi:10.1093/nar/gkv652)
Supplement: SUPPLEMENTARY DATA [file supp_43_14_7021__index.html]

Human DNA ligase III bridges two DNA ends to promote specific intermolecular DNA end joining — SUPPLEMENTARY DATA 

# Human DNA ligase III bridges two DNA ends to promote specific intermolecular DNA end joining

## SUPPLEMENTARY DATA

- SUPPLEMENTARY DATA
