# Supplementary material for: Human DNA ligase III bridges two DNA ends to promote specific intermolecular DNA end joining
Source: Nucleic Acids Res. 2015 Jun 29;43(14):7021–31. doi: 10.1093/nar/gkv652 (PMC4538836; doi:10.1093/nar/gkv652)

**Supplementary Table 1.** Apparent DNA binding affinities of LigIII proteins used in this study. DNA binding activity was measured in TR-FRET buffer containing 80 mM NaCl by fluorescence polarization. A FITC labelled DNA was used to form a 1:1 complex of LigIII bound to a DNA duplex.

| | **$K_{app}$ (nM)** |
|---|---|
| LigIII | 155 ± 4 |
| ΔZnF LigIII | 166 ±20 |
| ΔOBD LigIII | 308 ±13 |

**Supplementary Table 2.** Structural parameters for LigIII and DNA complexes from SAXS data

| **SAXS sample** | **$D_{max}$ (Å)** | **$R_g$[#] (Å)** |
|---|---|---|
| LigIII | ~198 | 59 |
| LigIII with DNA (3 nucleotide overhang) | ~200 | 59 |
| LigIII with a nicked DNA duplex | ~200 | 59 |
| ΔZnF LigIII | ~130 | 38 |
| ΔZnF LigIII with DNA (3 nucleotide overhang) | ~117 | 34 |
| ΔZnF LigIII with a nicked DNA duplex | ~115 | 33 |
| ΔOBD LigIII | ~141 | 46 |
| ΔOBD LigIII with DNA (3 nucleotide overhang) | ~130 | 43 |
| ΔOBD LigIII with a nicked DNA duplex | ~125 | 40 |

[#] $R_g$ is the radius of gyration calculated from the Guinier approximation. $D_{max}$ is the maximum dimension of the protein estimated from the pair distribution (*cf*. Figure 6).

**Supplementary Figure 1:** Positive and Negative controls for the TR-FRET DNA bridging assay. **(A)** An intact DNA duplex (DNA1) labeled with $Tb^{3+}$ and FITC on both 5' ends served as a standard to determine the maximum TR-FRET signal generated by our assay conditions. Dually labeled DNA1 (10 nM) was mixed with unlabeled DNA (500 nM) to mimic standard assay conditions. This sample produced a constant TR-FRET signal of 3.0, approximately 1.5- to 2-fold higher than the TR-FRET signal generated in a DNA bridging assay, which uses a limiting concentration of LigIII (see Figure 2 and Methods). Dual labeled, nicked DNAs (DNA2, DNA3) showed intermediate values of TR-FRET (2.0 to 2.5) that may reflect differences in the labeling efficiencies for these DNAs. **(B)** Human DNA ligase 1 (100nM) failed to generate a TR-FRET signal and lacks the zinc finger domain that is required for DNA bridging by LigIII.

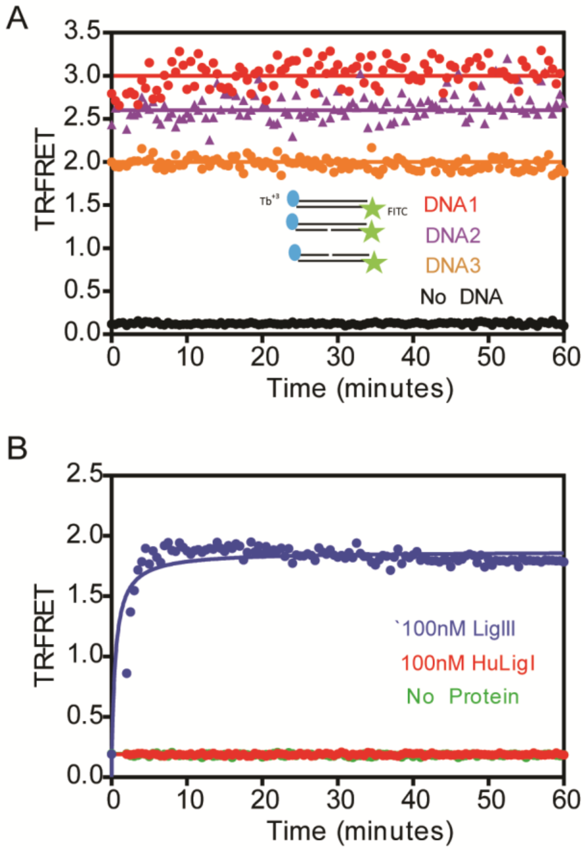

**Supplementary Figure 2:** Dissociation of labeled DNAs from the bridging complex. Addition of an unlabeled competitor DNA resulted in the loss of the TR-FRET signal with an apparent rate of 0.1 $min^{-1}$ in the presence of a saturating amount of competitor DNA.

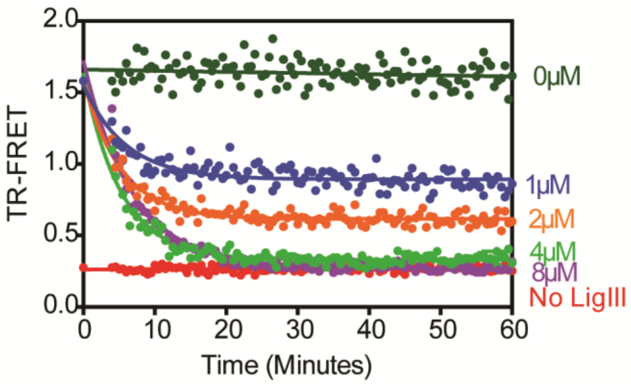


**Supplementary Figure 3:** Pull-down of blunt-ended DNAs in a bridging complex. The pull-down assay was performed as described in the Methods, using a pair of blunt-ended DNAs. LigIII forms a bridging complex with blunt-ended DNAs in a manner similar to the complex with three-nucleotide overhangs shown in Figure 8.

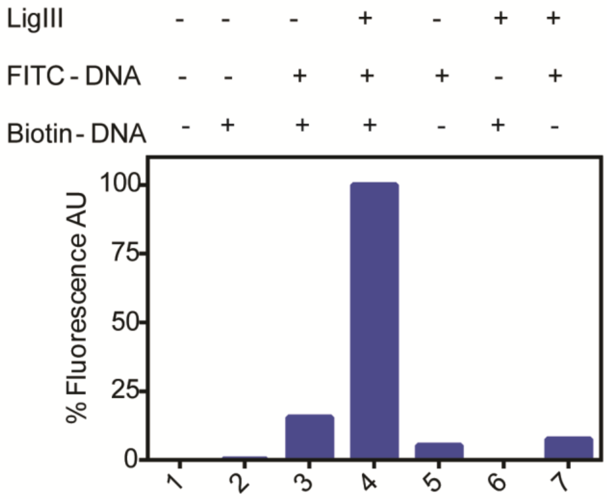

**Supplementary Figure 4:** DNA binding of adenylated and deadenylated LigIII. FITC labelled dsDNA (25 nM) or a nicked DNA duplex (25 nM) was titrated with increasing concentrations of (**A**) adenylated LigIII or (**B**) deadenylated LigIII. The formation of a protein-DNA complex is indicated by an increase in fluorescence anisotropy. Adenylated and deadenylated LigIII show comparable DNA binding activity.

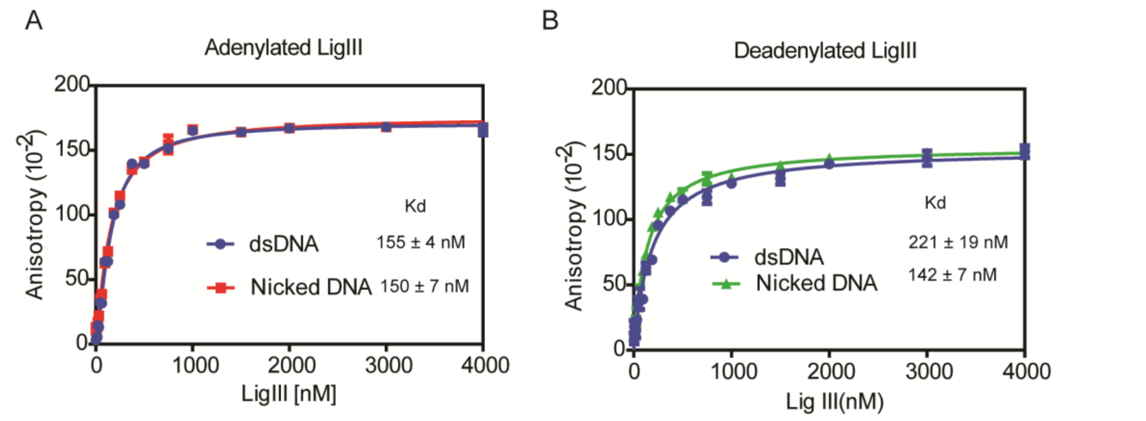


**Supplementary Figure 5:** Intermolecular DNA ligation by LigIII. The time course of DNA ligation was monitored for substrates with complementary 3-nucleotide overhangs (**A**) or with blunt ends (**B**). The rate of blunt end ligation catalyzed by LigIII is significantly slower than the ligation of DNAs with complementary overhangs.

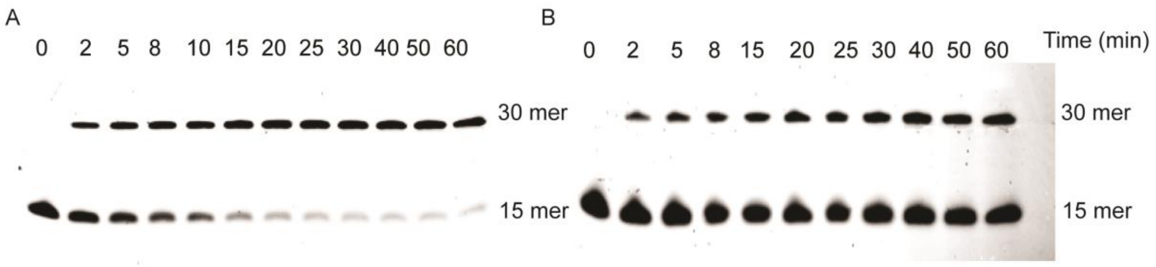

Supplement: SUPPLEMENTARY DATA [file supp_gkv652_nar-00686-h-2015-File010.pdf]
